# Autogenous Periosteal Graft Along with Open Flap Debridement Versus Open Flap Debridement Alone for the Treatment of Grade II Furcation Defect in Chronic Periodontitis Patients: A Systematic Review and Meta-Analysis

**DOI:** 10.3390/medicina61050905

**Published:** 2025-05-16

**Authors:** Swapna A. Mahale, Prasad Dhadse, Sumedha Thosar, Vedant Bhandari, Akhil Patil, Sadatullah Syed, Ranjeet Ajit Bapat, Tanay Chaubal, Sumaiya Zabin Eusufzai, Shahabe Saquib Abullais

**Affiliations:** 1 Department of Periodontology, Sharad Pawar Dental College and Hospital, Datta Meghe Institute of Higher Education & Research, Wardha 442001, India; prasad.perio@dmiher.edu.in; 2 Department of Periodontology, MGV’s KBH Dental College and Hospital, Nashik 422003, India; sumedhathosar@gmail.com (S.T.); thevedb99@gmail.com (V.B.); akhilpt1214@gmail.com (A.P.); 3 Department of Diagnostic Sciences and Department of Dental Education, College of Dentistry, King Khalid University, Abha 62521, Saudi Arabia; sasadat@kku.edu.sa; 4 Restorative Dentistry Division, School of Dentistry, IMU University, Kuala Lumpur 57000, Malaysia; ranjeetajit@imu.edu.my; 5 College of Dentistry, University of Oklahoma Health Sciences Center, Oklahoma City, OK 73104, USA; tanay-chaubal@ouhsc.edu; 6 School of Dental Sciences, Universiti Sains Malaysia, Kota Bharu 16150, Malaysia; sumaiya@student.usm.my; 7 Department of Periodontics and Community Dental Science, College of Dentistry, King Khalid University, Abha 62521, Saudi Arabia; sshahabe@kku.edu.sa

**Keywords:** grade II furcation defect, open flap debridement, autogenous periosteal graft, meta-analysis, systematic review

## Abstract

*Background and Objectives*: Periodontal regeneration involves techniques intended at restoring the lost supporting tissue around a periodontally weakened tooth. These regenerative methods frequently utilize periosteal grafts to stimulate the evolvement of vital adjacent tissues. This paper intended to evaluate the use of autogenous periosteal grafts in treating grade II furcation defects (Glickman Classification 1953) in patients with chronic periodontitis. *Materials and Methods*: The databases MEDLINE (via PubMed), Cochrane, EBSCO, and Google Scholar were searched for papers published in English from January 1991 till December 2022. Three individuals examined the reclaimed articles according to the inclusion norms. Randomized controlled trials (RCTs) assessing the efficacy of autogenous periosteal grafts for treating Grade II furcation defects in chronic periodontitis patients were involved. Only four related studies were identified for data extraction, involving 80 patients aged 18 to 52 years. Outcome variables measured included horizontal bone loss (HD), vertical bone loss (VD), pocket depth (PD), clinical attachment level (CAL), bone height (BH), gingival recession (GR), plaque index (PI), and gingival index (GI). Data were examined using RevMan 5.4.1 software. Mean differences and 95% confidence intervals were employed to estimate effect sizes. *Results*: Both groups showed similar results for reductions in PI, GI, and BOP. However, The periosteal graft also yielded better outcomes for CAL gain, BH, and GR. The meta-analysis showed a significant overall effect of Periosteal Barrier Membrane (PBM) on horizontal and vertical bony change levels, but subgroup differences between unilateral and bilateral applications were not statistically significant due to high heterogeneity. Although the bilateral subgroup demonstrated significant benefits of PBM treatment, the overall findings across the clinical attachment level group remain inconclusive. *Conclusion:* Current evidence suggests that while PBM may benefit bilateral mandibular sites, and autogenous periosteal grafts offer no added advantage over OFD alone in Grade II furcation defects, the overall findings remain inconclusive.

## 1. Introduction

Periodontal regeneration strives to restore damaged periodontal tissues, like bone, cementum, and the periodontal ligament, to their initial condition, using a combination of mechanical and surgical techniques [1]. The therapeutic strategies aim to either reduce or eliminate the pockets that act as reservoirs for pathogenic microorganisms [2,3]. The new attachment procedure focuses on restoring lost periodontal tissues with the advent of guided bone regeneration (GBR) with barrier membranes, which have a synergistic effect, enhancing regenerative outcomes [4,5,6].

Based on the biological principle of selective cell repopulation, the Guided Tissue Regeneration (GTR) technique hinders the growth of long junctional epithelium and encourages periodontal regeneration by excluding gingival epithelium apical migration and enabling the relocation of cells from the periodontal ligament and bone near the defect [7]. A wide variety of synthetic and naturally derived materials and absorbable and non-absorbable membranes were explored as potential barrier membranes in clinical practice. Current barrier membranes have shown some degree of effectiveness in regenerating interproximal defects [8,9]. The continuous need for improved effectiveness of barrier membranes has fostered the researchers’ idea of developing newer biomaterials. Selecting the most appropriate membrane for clinical use necessitates an in-depth understanding of its composition, as well as factors such as biodegradability [10].

For GTR, an array of non-absorbable materials was employed, comprising expanded polytetrafluoroethylene (ePTFE) and millipore membrane [11,12]. The primary benefit of non-absorbable barrier membranes is their ability to offer the appropriate gap beneath the barrier where such a compartment might not otherwise be achievable. However, the removal of the membrane necessitates a second surgical treatment, adding to the subject’s and operator’s distress. Considering this, investigations on humans and animals have assessed absorbable GTR membranes like collagen, ethyl cellulose, polylactic acid, and calcium sulfate for the GTR technique [13,14]. Other drawbacks of absorbable membranes are their lack of stiffness and embedded support structure.

According to a selection of studies, absorbable and non-absorbable membrane groups did not differ in their clinical attachment level (CAL) gain [15]. The usefulness of GTR for class II furcation defects treatment outperforms open flap debridement (OFD), which leads to a decline in the pocket and furcation depth and has shown improvements in horizontal and vertical attachment levels. However, these advancements have been modest and inconsistent [16].

Because autogenous periosteum with a layer of connective tissue satisfies the criteria for an ideal material and is biologically acceptable, some researchers attempted to address this issue by employing it in place of the current barrier membrane [17,18,19].

Human periodontal intrabony and furcation deficiencies have been studied in relation to the combined effects of barrier membrane and bone grafting as part of a combined periodontal regenerative therapy (CPRT) [20]. The incorporation of filler materials beneath the barrier membrane promotes the coronal migration of progenitor cells by acting as a physical barrier and preventing membrane collapse [21,22]. Clinical results from CPRT with autogenous periosteal membranes and bone grafts have been encouraging when contrasted with open-flap debridement or GTR without any filler material. However, human histologic evidence demonstrating the usefulness of autogenous periosteal membranes, either by themselves or in conjunction with bone transplants, is still lacking [19,23].

The periosteum has long been recognized for its essential role in bone repair and regeneration [24]. When used as a graft or barrier, periosteal tissue can effectively resolve interproximal periodontal defects [17]. Since autogenous periosteal grafts fulfill the criteria of an ideal biomaterial and are well-tolerated by the body, they present an appealing alternative to current barrier membrane materials [25].

Two tissue layers make up the periosteum: the inner cambial region, which comprises undifferentiated mesenchymal cells and osteogenic progenitor cells that aid in bone production, and the outside fibroblast layer, which gives attachment to soft tissue. Periosteal cells can produce an extracellular matrix and a membranous structure under certain circumstances [26]. In trials involving both humans and animals, the periosteum was utilized as a graft material to promote bone growth. Free periosteum grafts from rabbits’ tibias were able to display the formation of bone and cartilage when implanted into the kidney’s capsule and the eye’s anterior chamber. When periosteal grafts were inserted into the rabbit mandible’s midline sutures, bone growth was observed [27]. In clinical trials, the advantage of periosteum in furcation defects [28], interproximal defects [18], gingival recession [29], and peri-radicular area [30] exhibited substantiation of bone fill as well as a decline in pocket depths and CAL.

Autogenous periosteal membranes have the ability to promote new bone production, decrease adverse tissue responses during recovery, and only require one surgical treatment. These periosteal membrane characteristics were taken into account when planning the current investigation to assess the periosteum’s effectiveness as a barrier membrane in furcation defects [31].

## 2. Materials and Methods

### 2.1. Enrolment of Protocol

**Figure 1:**
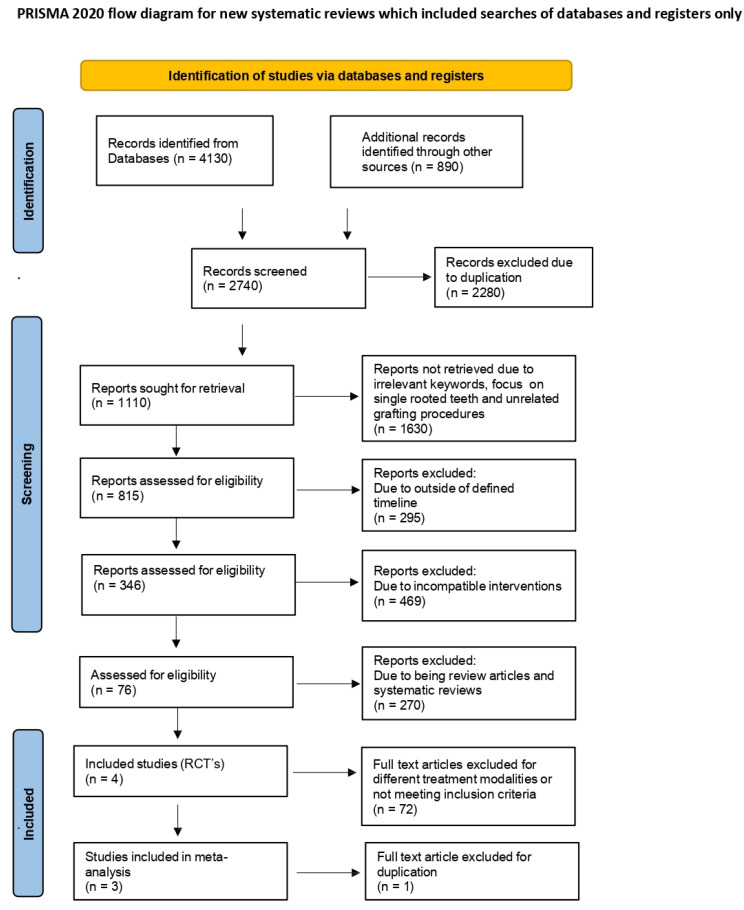
Prisma Flow chart.

The review process was added to PROSPERO (CRD42022314355/Dt-8/4/2022), the global database of listed systematic reviews, to obviate any unintentional duplication of the research on this subject. The research paper has been prepared as per the PRISMA statement and the Cochrane Handbook for Systematic Reviews of Interventions, as well as the Preferred Reporting Items for Systematic Review and Meta-analysis (PRISMA) recommendations [32]. The PRISMA flow chart illustrated in (Figure 1) outlines the process of article screening, from the initiation of the review to the thorough evaluation of the papers. “What is the viability of autogenous periosteal membrane in conjunction with open flap debridement and open flap debridement alone in the treatment of grade II furcation defects in patients with chronic periodontitis?”.

### 2.2. Focused PICOS Question

For this review, the PICOS model listed below was used: 

P—Chronic periodontitis patients with Grade II furcation defects.

I—The intervention involved the use of an autogenous periosteal graft in conjunction with open flap debridement.

C—The conventional open flap debridement surgical procedure was used for comparison.

O—The many outcomes evaluated included the following.

Primary outcomes:

1. Probing pocket depth (PPD).
2. Clinical attachment level (CAL).
3. Horizontal dimension of the furcation defect.
4. Vertical dimension of furcation defect.
5. Radiographic measurement of bone height (BH).

Secondary outcomes:

1. Plaque Index.
2. Gingival Index.
3. Sulcular bleeding index.
4. Gingival recession.
5. Tooth mobility.
6. Transmission electron microscopy (TEM) for histological changes.

All these clinical indicators were assessed for at least six months in the included studies.

S—Randomized controlled clinical trials (RCTs) that were limited to grade II furcation defects and literature published exclusively in English were searched.

### 2.3. Search Strategy

A thorough literature review was conducted from January 1991 until December 2022. A variety of search strategies were used to look through the information gathered for this systematic review. Four online databases, i.e., EBSCO, SCOPUS, Google Scholar, and MEDLINE, were searched for published publications as shown in Table 1. A thorough search method was developed for MEDLINE. Searches were combined using Boolean operators (AND and OR), and the body of evidence was explored via MeSH terms, keywords, and other free terms. ((open flap debridement OR root debridement)) AND (periosteum as barrier membrane OR periosteal pedicle graft) (class II furcation deficiency, a class II furcation involvement, or a grade II furcation involvement) ((patients with chronic periodontitis, periodontal disorders, periodontal surgery, or periodontitis)) AND ((RCT, or randomized controlled trial)).

The OpenGray database and the bibliography of all possible papers were thoroughly inspected in order to locate possibly related unpublished research or papers that were not available through electronic searching. Journal of Clinical Periodontology, Journal of Indian Society of Periodontology, Journal of Periodontology, and Journal of Periodontal Research were the four dental journals whose online databases were also analyzed. The article search was performed without any time limit. However, the systematic review covered human randomized controlled trials that were published in English. The publications were hand-searched using reference lists that may have been overlooked during the initial search based on inclusion criteria.

### 2.4. Inclusion Criteria and Exclusion Criteria

The titles and abstracts of the studies were used to filter them; if this information was insufficient to draw a conclusion, the entire articles were assessed. For inclusion, randomized controlled trials that lasted at least six months were deemed suitable. Research presenting outcomes from patients with chronic periodontitis and Grade II furcation involvement who underwent autogenous periosteal graft after open flap surgery were taken into consideration.

Literary works were accepted if they satisfied the following requirements.

Inclusion criteria:

1. Chronic periodontitis patients.
2. Patients of more than 18 years of age.
3. Healthy patients without any systemic disease.
4. Class II buccal/facial furcation defect in molars as per Glickman’s classification.
5. Probing pocket depth (PPD) must be 5 mm or more.

Exclusion criteria:

1. Patients with furcation involvement in the third molars were excluded.
2. Smoking patients and former smokers were excluded.
3. Pregnant and lactating females were excluded.
4. Patients who underwent treatment prior to the study.
5. Patients on oral contraceptives.
6. Non-compliant patients with no oral hygiene maintenance schedule.

### 2.5. Screening and Data Extraction

Every reviewer executed an independent screening of the papers’ titles and abstracts primarily. Papers that did not fit the criteria for selection were not retained. Duplicates were exempted with the help of Mendeley Desktop version 1.19.8. Reviewers checked every relevant paper carefully to determine if it fulfilled the criteria for inclusion in acquiring full-text versions. Discussions were used to sort out any conflicts.

Papers that failed to comply with the inclusion criteria were discarded. They rationalized their exclusion by presenting the justifications. A Microsoft Excel sheet was created by extracting the data from the included research papers. 

### 2.6. Measurement of Outcome

Primary Outcome:

- Clinical attachment level (CAL) is the distance from the cementoenamel junction (CEJ) to the base of the periodontal pocket. Pocket depth (PD) was assessed from the free gingival margin to the base of the periodontal pocket after one, two, three, four, six weeks, two months, and six months of follow-up examination. An increase in CAL indicates a loss of attachment, while a decrease in CAL indicates a gain of attachment.
- Pocket depth (PD) is a pathologic fissure between a tooth and the crevicular epithelium which is limited at its apex by the junctional epithelium. Change in the clinical attachment level (CAL) was calculated as the distance between the CEJ and the base of the periodontal pocket.
- Variation in bone defect fill (vertical and horizontal bone defect) was depicted as a greater bone density on the radiograph, which was noted at baseline and 6 months
- An increase in bone opacity from baseline to 6-month on radiographs indicated a change in bone defect fill (bone height).

Secondary Outcome:

- A decrease in plaque was related to a change in the Plaque Index at the 6-month recall.
- A decrease in gingival inflammation was related to a change in the Gingival Index at the 6-month recall.
- Change in gingival recession was measured from the gingival marginal to CEJ at different time intervals.
- Change in the sulcular bleeding index at different time intervals.
- Tooth mobility was measured at different time intervals.
- Transmission electron microscopy (TEM) for histological changes was evaluated post-operatively at 6 months follow-up.

### 2.7. Study Selection

A comprehensive database search identified 5020 records using keywords related to periosteal graft and Grade II furcation defects. Of these, 4130 records were retrieved using general keywords such as open flap debridement, furcation defect, and chronic periodontitis. An additional 890 papers were identified through expanded keyword combinations involving periosteal graft, Grade II furcation defect, and intrabony defect, bringing the total to 5020. After removing 2280 duplicate records, 2740 unique studies were screened. Of these, 1630 papers were excluded based on irrelevant keywords, or because they focused on single-rooted teeth and unrelated grafting procedures. This narrowed the pool to 1110 studies, which were further reduced to 815 after eliminating those outside the defined timeline (1991–2022). Subsequently, 469 papers were excluded due to incompatible interventions or non-qualifying methodologies. This resulted in 346 articles being eligible for full-text review. Among these, 270 were excluded as they were review articles or systematic reviews. The remaining 76 articles were evaluated, of which 72 were excluded for different treatment modalities or for not meeting inclusion criteria. Ultimately, 4 randomized controlled trials were initially selected, and after removing one duplicate, 3 studies were finalized for inclusion in the systematic review and meta-analysis (Figure 1).

### 2.8. Risk of Bias

**Figure 2:**
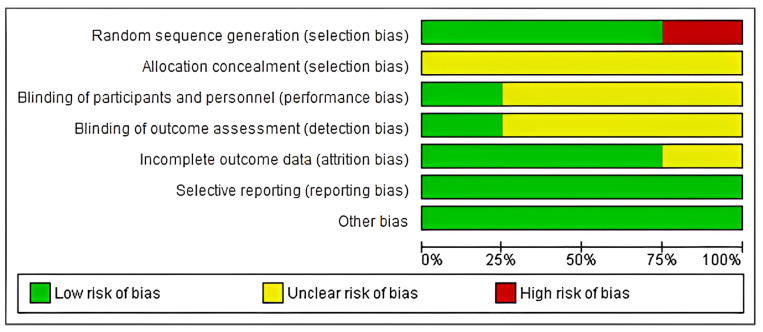
Risk of bias graph.

By means of the risk of bias valuation tool (a technique developed by the Cochrane Collaboration), three investigators (S.M, P.S., and S.T) independently examined the quality of selected studies [32]. Any disputes over the review were resolved through mutual discussion. The readings were characterized as a high, low, or unclear risk of bias for the incorporated studies were grouped into (as shown in Figure 2):

(1) low risk: when all criteria were met, or one criterion was unclear/not met;
(2) moderate risk: when two criteria were unclear/not met;
(3) high risk: when more than two criteria were not met.

## 3. Results

The systematic review mainly incorporated four publications. The information on the risk of bias valuation is presented in Table 2, while general study features are presented in Table 3, data taken from the chosen articles is listed in Table 4.

**Figure 3:**
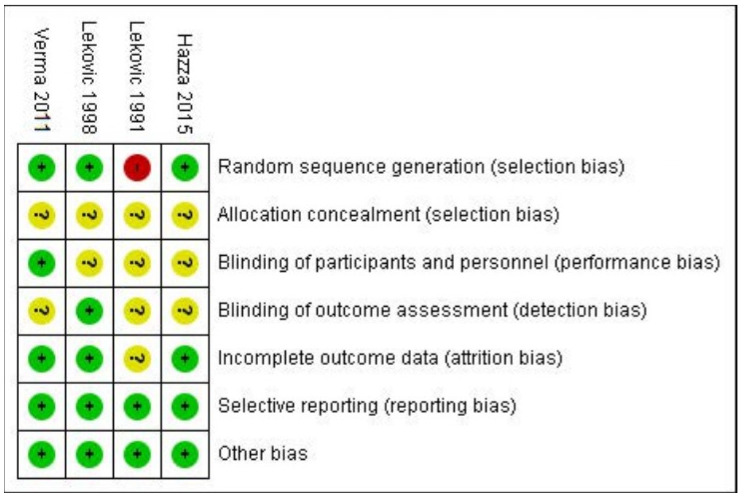
Risk of bias summary [28,33,34,35].

Among the incorporated studies, two (Lekovic 1998, Verma 2011) showed a low risk of bias [33,34]; a study by Hazzaa 2015 disclosed moderate risk [35], and a study by Lekovic 1991 [28] indicated a high risk of bias, as depicted in Figure 3. As details of randomization, allocation concealment and blinding were not mentioned by Lekovic 1991 [28], which gave rise to a high risk of bias.

**Table 2:** Risk of bias assessment according to RoB-2 tool.

Sr. No.	Study ID	Random Sequence Generation	Allocation Concealment	Blinding of Participants and Personnel	Blinding of Outcome Assessment	Incomplete Outcome Data	Selective Reporting	Other Bias	Risk of Bias
1.	Lekovic 1991 [28]	High	Unclear	Unclear	Unclear	Unclear	Low	Low	High risk
2.	Lekovic 1998 [34]	Low	Unclear	Unclear	Low	Low	Low	Low	Low risk
3.	Verma 2011 [33]	Low	Unclear	Low	Unclear	Low	Low	Low	Low risk

**Table 3:** General study characteristics.

Study ID	Study Design	Sample Size	Age Group	Gender	Diagnosis	Inclusion Criteria
Lekovic 1991 [28]	clinical—split mouth study	15	-	8 males, 7 females	Periodontitis with class 2 furcation defect in lower molars	Two Class II furcation involvements on the lower molar teeth’s facial side in adults furcation with >5 mm pocket depth after phase-I therapy.
Lekovic 1998 [34]	clinical split mouth study	28	-	-	Each side of the jaw has class II mandibular buccal furcation deformities.	(1) The presence of two Class II mandibular buccal furcation defects on each side of the jaw; radiography and clinical evaluation were used to diagnose the Class II furcation defect. (2) Both roots’ viability tests (cold and electrical pulp tests) reported positive. (3) The buccal aspect of the tooth has keratinized gingiva that is at least 2 mm thick.
Verma 2011 [33]	clinical split mouth study	11 (initially 12 patients were considered) 12/12	28–49 years	7 males, 5 females	Periodontitis with grade 2 furcation involvement in lower molars bilaterally.	Patients having at least one pair of bilateral buccal grade II furcation defects of lower molars Selected subjects were not under any medication during 1st month before surgery. Periodontal probing depth (PPD) at mid furcation area was 5 mm or more. Patients had good oral hygiene. Patients were free of any systemic disease.
Hala Hazzaa 2015 [35]	Clinical parallel design	26	37–52 years	15 females, 11 men	Chronic periodontitis along with class II buccal furcation defect in a mandibular molar.	(1) The mandibular molar has a class II buccal furcation defect, as defined by Glickman’s classification (1953). (2) There are no systemic disorders that could affect the therapy’s success, as determined by the modified Cornell medical index. (3) Adherence to plaque control guidelines following initial therapy, using the plaque index values (0 or 1) according to Silness and Löe (1964). (4) Vertical probing pocket depths (VPD) of ≥5 mm and clinical attachment levels (CAL) ≥4 mm four weeks following initial therapy. (5) Gingival margin positioned coronally to the furcation fornix.

**Table 4:** Data extraction from the included studies.

Reference	MD in Horizontal Bone Loss	MD in Vertical Bone Loss	MD in Clinical Attachment Level	MD in PPD	MD in Gingival Recession	MD in Plaque Index	MD in Sulcular Bleeding Index	MD in Gingival Index
Lekovic et al. 1991 [28]	1.60 ± 0.63	2.00 ± 0.54	2.40 ± 1.35	4.13 ± 0.92	0.73 ± 0.88	0.11 ± 0.24	0.27 ± 0.46	-
Lekovic et al. 1998 [34]	1.60 ± 0.21	1.93 ± 0.15	2.71 ± 0.40	3.66 ± 0.24	1.07 ± 0.37	0.14 ± 0.05	0.02 ± 0.04	-
Verma et al. 2011 [33]	1.50 ± 0.55	1.67 ± 0.49	2.17 ± 0.72	-	-	-	-	0.83 + 0.19
Hazzaa et al. 2015 [35]	59.7 ± 13.4	60 ± 8.6	63.7 + 8.8	-	-	-	-	

### 3.1. Study Characteristics

The details of the four studies that were part of this systematic review are listed in Table 1. Every study that was part of the collection was a randomized controlled clinical trial. One study (Hazzaa 2015 [35]) showed a parallel study design, while the remaining three studies (Lekovic 1991, Lekovic 1998, and Verma 2011 [28,33,34]) showed a split-mouth design. These studies were conducted in different parts of the world—two in Yugoslavia, one in India and one in Egypt.

In all the included studies, the furcation defect was covered with periosteal membrane graft in the experimental group and no periosteal membrane in control group. A total of 81 participants with ages varying from 28 to 52 years were appraised. The age of participants. All the study participants were diagnosed with Class II furcation defect in the mandibular molar region. The primary outcomes were analyzed as probing pocket depth (PPD), clinical attachment levels (CAL), horizontal dimension of the furcation defect, vertical dimension of furcation defect, radiographic measurement of bone height (BH).

Secondary study measures were the Plaque Index, Gingival Index, Sulcular bleeding index, Gingival recession, Tooth mobility, Transmission electron microscopy (TEM) for histological changes. In all the studies, a follow-up period of 6 months was carried out. Table 2 provides an overview of the extracted data and an illustration of the features of the included papers.

In all the trials, non-surgical therapy was performed under local anesthetic, along with occlusal adjustment, except in one study, Lekovic et al. [28], where occlusal adjustment was not mentioned.

Two studies [28,33] assessed open flap debridement along with the periosteal graft in the intervention group and only open flap debridement in the control group. This study [34] evaluated the use of a coronally positioned flap along with a periosteal graft with only a coronally positioned flap. In another study, ref. [35], they compared open flap debridement+ periosteal graft only to open flap debridement and open flap debridement+ periosteal graft + DFDBA graft. All the trials had a recall visit of 6 months.

In one of the studies, ref. [28], the participants were prescribed oral antibiotics for 7 days (penicillin VK 250 mg q.i.d) and oral analgesics (ibuprofen) as needed. Patients in a different study received 500 mg of penicillin VK q.i.d. for seven days. Patients with penicillin allergies were treated with 250 mg of erythromycin sterate every three months. Patients were advised to take 800 mg of Ibuprofen t.i.d. as needed as an analgesic [34]. Amoxicillin 500 mg t.i.d. for 7 days and Nimesulide 100 mg b.d for 5 days were recommended as an antibiotic regime of in another study [33]. Patients who were suffering discomfort following surgery were given analgesics (400 mg of ibuprofen). The patients were put on an infection control regimen that included systemic antibiotic treatment for seven days (two grams of amoxicillin–clavulanic acid per day) [35].

In all the studies [28,34,35], 0.12% chlorhexidine gluconate mouthwash for chemical plaque control was advised for the participants, except in one study [33] where 0.2% chlorhexidine mouthwash was prescribed.

Chemical plaque control was prescribed for 2 weeks in one study [28], 3 weeks in another study [34], and for 6 weeks in the third study, [35] whereas the time period was not specified in the fourth study [33].

Lekovic et al. [28] used a periodontal probe and the Michigan probe was used by Lekovic et al. [34]. Verma et al. [33] used the UNC-15 probe and Hala Hazza et al. [35] used the Williams probe for the measurement of periodontal pockets. All the studies used a custom-made grooved acrylic stent for the reproducibility of probing pocket depths.

With the exception of the Hazza et al. study [35], which removed the sutures at two weeks, all trials performed suture removal at one week.

### 3.2. Statistical Analysis

The Higgins index and chi-square were used to determine the heterogeneity following the Cochrane Handbook [I2]. A meta-analysis was conducted using RevMan 5.4.1 software to compare horizontal bone changes, vertical bone changes, and clinical attachment level changes with or without PBM (Periosteal Barrier Membrane) intervention. Out of the four included studies, three studies have split mouth design, and one study has a parallel arm design. So, for meta-analysis, the parallel arm design (Hazza et al. 2015 [35]) was excluded.

Effect-size metric: We used the Standardized Mean Difference (SMD) calculated by RevMan 5.4.1, which applies Hedges’ g by default to correct for small sample bias, especially when study sample sizes are unequal or small.

Model estimator: A random-effects model was employed using the DerSimonian and Laird (DL) method, which is the default estimator for between-study variance (τ^2^) in RevMan 5.4.1. While RevMan does not offer alternative estimators such as REML (Restricted Maximum Likelihood), the DL method remains widely accepted for clinical meta-analyses.

Heterogeneity assessment: Heterogeneity was assessed using the Tau^2^ statistic, Chi^2^ test (Cochran’s Q), and I^2^ statistic. I^2^ values > 50% were considered indicative of substantial heterogeneity.

Subgroup analyses: Subgroup analyses were conducted within RevMan 5.4.1 by grouping studies based on unilateral vs. bilateral mandibular molar involvement. Differences between subgroups were evaluated using the Chi^2^ test for subgroup differences provided by the software, along with its associated I^2^ to gauge the consistency of subgroup differences. The pooled effect size, considered as a standardized mean difference (SMD), revealed a significant overall effect. Due to high heterogeneity, subgroup meta-analysis has been performed.

### 3.3. Horizontal Bone Change

The meta-analysis provided below was applied to evaluate the impact of PBM (Periosteal Barrier Membrane) on horizontal bone level changes. Data from three studies have been analyzed as they meet the inclusion criteria of PICOS: Lekovic 1991, Lekovic 1998, and Verma 2011 [28,33,34]. A subgroup analysis was conducted to assess whether the effectiveness of the intervention differed between unilateral and bilateral mandibular molar teeth involvement.

The unilateral subgroup, represented by the studies Lekovic 1991 and Lekovic 1998 [28,34], showed a large positive treatment effect. However, the effect was not statistically significant (Z-value not provided but implied to be non-significant), and high heterogeneity was observed (Tau^2^ = 22.67; Chi^2^ = 24.25, df = 1, *p* < 0.00001; I^2^ = 96%). This suggests substantial variability between the two studies and indicates that the results should be interpreted with caution. Although the observed effect was substantial in magnitude, the high inconsistency reduces the reliability of the pooled estimate in this subgroup.

In contrast, the bilateral subgroup, represented by the Verma 2011 study [33], demonstrated a moderate but statistically significant treatment effect (Test for overall effect: Z = 4.01, *p* < 0.0001), with consistent findings (no heterogeneity reported, as it was a single study). This suggests that the intervention is effective when applied bilaterally, with more confidence due to the statistical significance and lack of heterogeneity.

When all studies were combined, the overall analysis showed a significant treatment effect (Z = 2.95, *p* = 0.003), but with high heterogeneity (Tau^2^ = 5.14; Chi^2^ = 26.00, df = 2, *p* < 0.00001; I^2^ = 92%), indicating substantial variation in effect sizes across studies.

**Figure 4:**
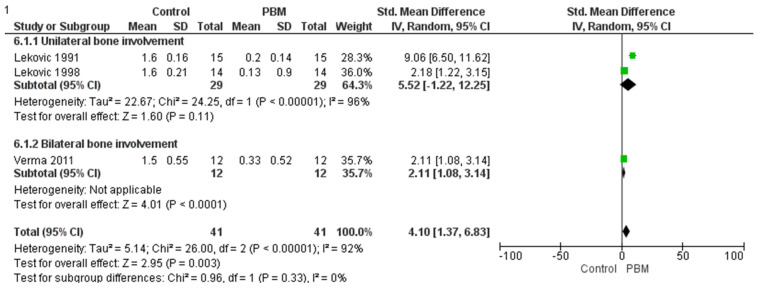
Forest plot for horizontal bone loss [28,33,34].

Importantly, the test for subgroup differences was not statistically significant (Chi^2^ = 0.96, df = 1, *p* = 0.33; I^2^ = 0%), indicating that the difference in treatment effect between unilateral and bilateral subgroups was not significant as shown in Figure 4. Therefore, while the unilateral subgroup showed a higher estimated effect, the statistical test does not support a conclusive difference between the two subgroups. Since diamond touches the line of no effect in the analysis of horizontal bone loss, this suggests that there is no statistically significant difference in bone level changes between the PBL group and the control group.

### 3.4. Vertical Bone Change Group

In this meta-analysis, we evaluated the effectiveness of Periosteal Barrier Membrane (PBM) treatment compared to the control across three studies, with subgroup analyses for unilateral and bilateral mandibular tooth involvement.

For the unilateral subgroup, the pooled effect size was not statistically significant (Z = 1.62, *p* = 0.11), with high heterogeneity observed (Tau^2^ = 31.92; Chi^2^ = 23.40, df = 1, *p* < 0.00001; I^2^ = 96%). This indicates substantial variability among studies, suggesting that the effect of PBM in unilateral mandibular teeth is inconsistent and inconclusive.

In contrast, the bilateral subgroup demonstrated a statistically significant positive effect of PBM treatment (Z = 2.46, *p* = 0.01), with no observed heterogeneity (I^2^ = 0%), indicating consistent findings across studies in this subgroup.

**Figure 5:**
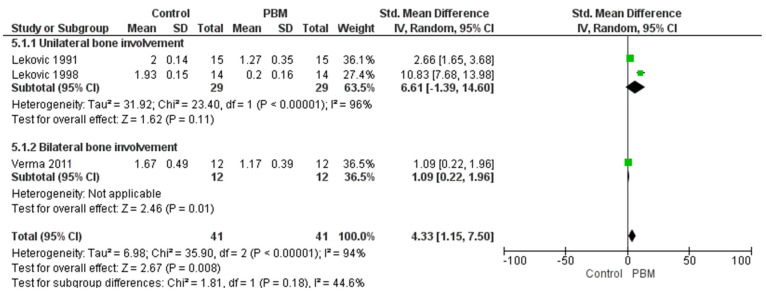
Forest plot for vertical bone loss [28,33,34].

The overall analysis, combining all studies, yielded a significant effect favoring PBM (Z = 2.67, *p* = 0.008), but with considerable heterogeneity (Tau^2^ = 6.98; Chi^2^ = 35.90, df = 2, *p* < 0.00001; I^2^ = 94%), reflecting variation in study outcomes as shown in Figure 5. The test for subgroup differences was not statistically significant (Chi^2^ = 1.81, df = 1, *p* = 0.18; I^2^ = 44.6%), suggesting that the difference in effectiveness between unilateral and bilateral treatments may not be substantial.

Overall, while PBM treatment appears to be effective, particularly in bilateral mandibular teeth, the high variability in the unilateral subgroup and across all studies indicates a need for further high-quality, standardized research to better understand the treatment’s impact in different clinical scenarios.

### 3.5. Clinical Attachment Level

**Figure 6:**
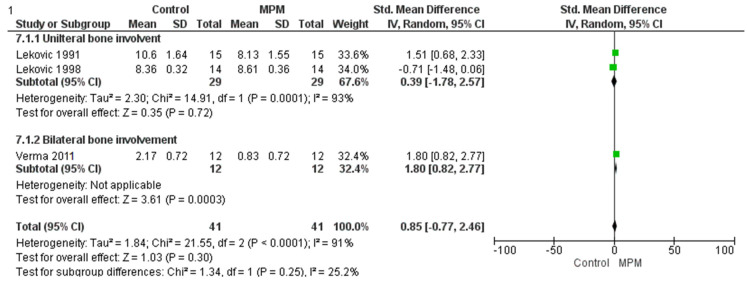
Forest plot for clinical attachment level [28,33,34].

A random-effects meta-analysis was conducted to assess the effect of Modified Periosteal Matrix (MPM) treatment on periodontal probing levels compared to control across three studies. Two studies (Lekovic 1991 and Lekovic 1998 [28,34]) focused on unilateral mandibular molar sites, while one study (Verma 2011) involved bilateral sites [33], allowing for subgroup analysis. In the unilateral subgroup, the overall effect was not statistically significant (Z = 0.77, *p* = 0.44) and showed considerable heterogeneity (Tau^2^ = 3.52; Chi^2^ = 20.78, df = 1, *p* < 0.00001; I^2^ = 95%), indicating substantial inconsistency between study results. In contrast, the bilateral subgroup (Verma 2011 [33]) demonstrated a statistically significant positive effect favoring MPM (Z = 4.56, *p* < 0.00001). However, the overall pooled analysis yielded a non-significant result (Z = 1.44, *p* = 0.15), with the diamond in the forest plot touching the line of no effect, meaning that the overall effect estimate includes the possibility of no difference between MPM and control. This result also exhibited substantial heterogeneity (Tau^2^ = 1.63; Chi^2^ = 41.73, df = 2, *p* < 0.00001; I^2^ = 95%), suggesting high variability across the included studies. Furthermore, the test for subgroup differences was not statistically significant (Chi^2^ = 0.04, df = 1, *p* = 0.84; I^2^ = 0%), indicating no meaningful difference in treatment effect between unilateral and bilateral applications as shown in Figure 6. In summary, while the bilateral subgroup alone showed significant benefits, the overall findings remain inconclusive due to statistical non-significance and high heterogeneity, warranting further research with more uniform study designs to better evaluate the efficacy of MPM.

## 4. Discussion

The management of furcation defects remains a critical issue in periodontal remedies due to the challenging location and irregular anatomy of the roots, which make biofilm elimination difficult with standard oral hygiene practices. Furcation involvement significantly surges the risk of tooth loss, influenced heavily by factors such as age, gender, smoking, and diabetes [36,37]. Surgical therapy options for these defects often comprise various reconstructive periodontal techniques and materials [38]. One such method is GTR, which uses barrier materials to promote bone regeneration and intertwining with new connective tissue in an effort to restore damaged periodontal tissues [38].

Studies on both humans and animals have revealed that periosteum, when utilized as a regenerative material, has the capacity to promote bone growth [27,39,40,41]. Barrier membranes employed in GTR allow periodontal ligament progenitor cells to rebuild tissues from the base of the defect while preventing epithelial downgrowth [42]. Autogenous periosteal grafts present a promising substitute to prevailing barrier membrane materials due to their biological compatibility and potential to meet the ideal material requirements. The periosteum is extremely vascular and rich in fibroblasts, osteoblasts, and stem cells [43]. These cells can develop into adipocytes, fibroblasts, osteoblasts, chondrocytes, and skeletal myocytes, which aid in the regeneration of bone, cementum, and periodontal ligament fibers [44].

In order to address grade II furcation deficits in patients with chronic periodontitis, this systematic review assessed open flap debridement both with and without autogenous periosteal grafts.

Randomized controlled trials were the main focus in order to guarantee methodological rigor and gather solid evidence. The most effective method for minimizing grade II furcation defects was the use of periosteal pedicle grafts as a barrier membrane [45].

Included searches involved chronic periodontitis patients with grade II furcation defects while excluding smokers, patients with systemic diseases, and those with third molars, as these factors negatively impact clinical attachment level gains, plaque reduction, and gingival recession outcomes following surgical interventions [46].

Since there was no statistically significant difference between the groups, the PI, SBI, and GI reductions had an equivalent outcome effect [28]. Though the change was not statistically significant, the findings for bone defect filling showed that the autogenous periosteal graft with the OFD was superior to coronally advanced flap or flap with DFDBA [47].

When autogenous periosteum was used as a barrier membrane, the results demonstrated the development of an osseous structure that provided sufficient bone fill for both vertical and horizontal defects [47].

The osteogenic potential of the periosteum, reviewed by Abu-Shahba et al. [48] suggests that the periosteum’s osteogenic potential contributes to enhanced bone regeneration, as it contains osteoprogenitor cells that aid in defect healing. This explains the significant changes in defect fill between the test and control groups, where the test group likely benefited from periosteal involvement or enhancement. According to Hirata et al., the statistically significant bone defect fill can be linked to the vascularized periosteum’s capacity to produce new bone [49]. Clinically, every study [17,28,33,35] that was part of this systematic review showed a significant enhancement in bone density. Because the autogenous periosteal graft was connected on one side to the mucoperiosteal flap, which promotes healing and preserves the essential cambium layer, which may promote bone formation, it retained its vascular supply in every study [50].

There was a statistically significant decrease in the depths of periodontal pockets in all studies examined for PPD. Infection and damage to the periosteum may arise if the pocket depth in the defect area reaches 5 mm or more, complicating the placement of the periosteal barrier membrane [33]. The membrane serves as a defending barrier, mitigating the likelihood of periosteal damage and infection while facilitating the proper alignment and stabilization of the healing tissue.

The analysis revealed a statistically significant change in clinical attachment level when a periosteal graft was utilized in conjunction with OFD as a barrier membrane. Regarding GR growth, one study [34] demonstrated evident results alongside OFD with autogenous periosteal graft, whereas another study [28] revealed no significant difference over a 6-month period. The heterogeneity in GR appears to result from the tendency of flaps to migrate apically throughout the healing process [17]. The pooled analysis demonstrated a mean difference of 1.6 mm in favor of the periosteal graft group for clinical attachment level gain. However, it is debatable whether a 1.6 mm gain represents a clinically significant improvement. Some studies suggest that a CAL gain of at least 2 mm is necessary to achieve meaningful improvements in periodontal stability and reduce the risk of further attachment loss [3].

In a study conducted by Hala Hazza et al. [35] transmission electron microscopy (TEM) was utilized to examine the gingival tissue. A correlation was identified between clinical and TEM data. OFD combined with autogenous periosteal grafts exhibited a significant presence of proliferating fibroblasts and ample collagen bundles. From a biological perspective, periosteal grafts’ protective effects may speed up healing by encouraging the growth and development of both native progenitor cells and those provided by the cambium layer [51].

The autogenous periosteal graft is advantageous due to its lack of requirement from a distinct surgical site for removal, facilitating straightforward harvesting and manipulation while minimizing further stress. It serves as a highly effective alternative to traditional barrier membranes in regenerative periodontal therapies, as it is a pedicle autograft that maintains its vascular supply. All studies in this review indicate that periosteal membrane placement markedly enhanced clinical and histological outcomes in the management of grade II furcation defects, particularly in lower molars, compared to control groups.

The requirement for histologic assessment to verify the effectiveness of the periosteal membrane in fostering periodontal regeneration may be one of the systematic review’s weaknesses. All of the studies had rather modest sample sizes. About six months were spent on the project. To create robust evidence, studies with longstanding recall visits are necessary. To obtain consistent outcomes from this method, future research should continue to follow up for three to five years. Only four publications made it into this meta-analysis and systematic review; many others were omitted if the criteria did not match or there was not enough data available. Other factors, including membrane tension, connective tissue thickness, periosteum viability, defect width, oral hygiene maintenance, and operator experience, may have a substantial impact on the outcomes of this systematic review.

This meta-analysis is limited by significant statistical heterogeneity, this high heterogeneity indicates that treatment effects varied substantially across studies, suggesting influences beyond random chance. Potential sources of this variability include differences in patient populations, periodontitis severity, surgical techniques, and CAL measurement methods. The small number of included studies hindered further exploration of these factors through subgroup analyses or meta-regression.

This heterogeneity has important implications for interpreting the findings. The pooled estimates represent an average effect and may not accurately reflect outcomes in specific clinical situations, thus limiting the generalizability of the results. Clinicians should therefore exercise caution when applying these findings to individual patients. While the analysis suggests potential benefits of periosteal grafts, the substantial heterogeneity prevents a definitive conclusion of consistent superiority over open flap debridement alone.

## 5. Conclusions

Therefore, given the study’s limitations, the findings of this systematic review and meta-analysis suggest that autogenous periosteal grafts in conjunction with open flap debridement (OFD) do not demonstrate a significant difference in outcomes compared to OFD alone for the treatment of Grade II furcation defects in chronic periodontitis. Furthermore, based on the meta-analysis, periosteal barrier membrane (PBM) treatment may show promising results for bilateral mandibular teeth involvement. However, no significant difference was observed for unilateral involvement, and the evidence remains inconclusive due to high variability among studies. Further studies with larger sample sizes and more consistent methodologies are needed to confirm these findings and provide a clearer picture of PBM’s effectiveness in different clinical scenarios. However, due to a lack of research with restricted data, a smaller population size, and 6-month recall visits, only a speculative conclusion can be made from this study. Therefore, to achieve a firm conclusion, higher-quality RCTs with a greater population size and longer recall visits are required.

## Figures and Tables

**Table 1:** Search Strategy.

Sr No	Database	Search Term
1	PUBMED	((Open flap debridement) OR (root debridement)) AND ((periosteum as barrier membrane) OR (periosteal pedicle graft)) AND (class II furcation deficiency, class II furcation involvement, OR grade II furcation involvement) AND ((Chronic Periodontitis(Other term)) AND ((RCT OR (Randomized Controlled Trial))
2	EBSCO	((Open flap debridement) AND (periosteum as barrier membrane) OR (periosteal pedicle graft)) AND ((Chronic Periodontitis)(Periodontitis)) AND (RCT)
3	Google Scholar	((Open flap debridement) OR (Periodontal Surgery) AND (periosteum as barrier membrane) OR (periosteal pedicle graft)) AND (grade II furcation) AND ((Chronic Periodontitis)(Periodontitis)) AND (RCT)
4	SCOPUS	((Open flap debridement) OR (Periodontal Flap Surgery) AND (periosteal pedicle graft)) AND ((grade II furcation) OR (Class II Furcation defect)) AND ((Chronic Periodontitis)(Periodontitis)) AND (RCT)

## Data Availability

The research data are available from the corresponding author and can be made available on request.

## Abbreviations

The following abbreviations are used in this manuscript:

GBR	Guided Bone Regeneration
GTR	Guided Tissue Regeneration
ePTFE	Expanded Polytetrafluoroethylene
CAL	Clinical Attachment Level
OFD	Open Flap Debridement
CPRT	Combined Periodontal Regenerative Therapy
PROSPERO	Prospective Register of Systematic Reviews
PRISMA	Preferred Reporting Items for Systematic Reviews and Meta-Analyses
PICOS	Population, Intervention, Comparison, Outcome, Study design
PPD	Probing Pocket Depth
BH	Bone Height
TEM	Transmission Electron Microscopy
RCT	Randomized Controlled Trial
MeSH	Medical Subject Headings
TEM	Transmission electron microscopy
CEJ	Cemento-enamel Junction
PBM	Periosteal Barrier Membrane
MPM	Modified Periodontal Management
